# Sutureless versus transcatheter valves for the treatment of aortic valve stenosis: a systematic review and meta-analysis

**DOI:** 10.1038/s41598-025-17857-7

**Published:** 2025-10-03

**Authors:** Lennert Minten, Marie Lamberigts, Leen Van Langenhoven, Peter Verbrugghe, Johan Bennett, Christophe Dubois, Bart Meuris

**Affiliations:** 1 Department of Cardiovascular Sciences, KU Leuven, Herestraat 49, Leuven, 3000 Belgium; 2 Department of Cardiovascular Medicine, University Hospitals Leuven (UZ Leuven), Leuven, Belgium; 3 Department of Cardiac Surgery, University Hospitals Leuven (UZ Leuven), Leuven, Belgium; 4 I-BioStat, KU Leuven & University of Hasselt, Leuven, Belgium

**Keywords:** Aortic valve stenosis, transcatheter aortic valve replacement, Sutureless aortic valve replacement, Systematic review, Meta-analysis, Valvular disease, Outcomes research

## Abstract

An important limitation of the pivotal randomized controlled trials that compared transcatheter aortic valve replacement (TAVR) to surgical aortic valve replacement (SAVR) is that the SAVR arm scarcely included sutureless bioprosthetic valves. We identified 13 retrospective propensity-matched studies in low (*n* = 2), intermediate (*n* = 8), and high-risk (*n* = 3) patients, using EuroSCORE and STS score to assess perioperative risk. One large registry drove the outcome in low-risk patients, showing better early survival with TAVR, lower rates of stroke and acute kidney injury. Intermediate-risk patients showed improved early and medium-term survival with SU-AVR, whilst in high-risk patients, no significant differences were seen between treatment options. Overall, across all risk categories, the rates of moderate and severe aortic regurgitation and permanent pacemaker implantation were significantly lower with SU-AVR, while transprosthetic gradients and duration of hospital stay were higher compared to TAVR. The differences in survival in the intermediate risk group are not in line with conclusions of pivotal randomized trials comparing TAVR with SAVR. Specific features of SU-AVR may account for these survival differences, positioning SU-AVR as a valid and safe alternative for patients at intermediate risk. Awaiting confirmation in randomized trials, careful patient selection and consideration of either of the AVR options in the heart team remain crucial.

**Supplementary Information:**

The online version contains supplementary material available at 10.1038/s41598-025-17857-7.

## Introduction

Degenerative aortic valve stenosis (AS) is the most prevalent heart valve disease in the western world, affecting 3% of the population above 75 years, and is one of the most frequently observed valve pathologies in daily clinical cardiology and cardiac surgery practice^1^. Surgical aortic valve replacement (SAVR) is the gold standard treatment for patients with symptomatic severe AS at low surgical risk below the age of 65 (American guidelines) or 75 (European guidelines)^2,3^. In contrast, patients > 80y (American guidelines) or ≥ 75y (European guidelines) should rather be considered for transcatheter aortic valve replacement (TAVR) when technically feasible. These recommendations leave room for an intermediate risk group (around 65-80y) in which both options are valuable alternatives. Final treatment choice is established through a process of shared decision making within the Heart Team, taking into account individual patient comorbidities, technical considerations, and importantly, patients’ preference. The guidelines do not specifically mention the use of sutureless or rapid deployment valves (RDV) for SAVR due to lack of randomized controlled trials (RCT) between sutureless aortic valve replacement (SU-AVR) and TAVR. In the search for innovative and less invasive alternatives to standard SAVR in a rapidly growing elderly population, another option has emerged besides TAVR (Central figure). This alternative avoids placement and tying of multiple sutures, and is referred to as sutureless aortic valve replacement (SU-AVR). The concept has existed for more than 50 years but the idea and the valves have been re-modernized^4^. Currently, two valve types are available: a sutureless valve called Perceval, and another valve that needs only 3 sutures, called Intuity (also referred to as RDV) The rapid-deployment Intuity Elite (Edwards Life Sciences, USA) works by a stainless steel cloth-covered frame that is delivered through balloon catheter expansion in the correct annular position^5^. The Perceval S or more recently the Perceval PLUS (Corcym, Italy, previously manufactured by Sorin group and LivaNova) utilizes a self-expanding nitinol frame that has a memory and deploys and positions the valve without the need for additional sutures^6^. Both approaches require a surgical incision, cardiopulmonary bypass (CPB), aortic cross clamping (AXC) and excision of the diseased calcified native aortic valve and debridement of the annulus. In comparison to the traditional stented aortic surgical prostheses, they do not need extensive suturing^7^. The main hypothesis is that faster implantation times may lead to shorter overall duration of the operation, with shorter CPB and AXC times, which is ideal in combination with minimally invasive access.

Because the sutureless valves, in their current form, are relatively new, there are few studies directly comparing TAVR with SU-AVR. The present systematic review therefore summarizes the available evidence and explores the potential benefit of SU-AVR over TAVR in patients at low, intermediate or high risk for SAVR.

## Methods

### Data sources and searches

We performed a systematic review of studies that compare TAVR and SU-AVR for the treatment of symptomatic severe AS. The electronic databases PUBMED, google scholar, LIMO and the Cochrane library were searched in October 2023. The MESH terms and appropriate free text was used: “sutureless aortic valve replacement”, “rapid deployment aortic valve replacement”, “surgical aortic valve replacement”, “transcatheter aortic valve implantation”, “transcatheter aortic valve replacement”, “minimally invasive aortic valve replacement”, “aortic stenosis”, “TAVI”, “TAVR”, “SAVR”, “AVR”, “AS”. The reference lists of articles found were also screened for relevance.

### Eligibility criteria and study selection

**Fig. 1:**
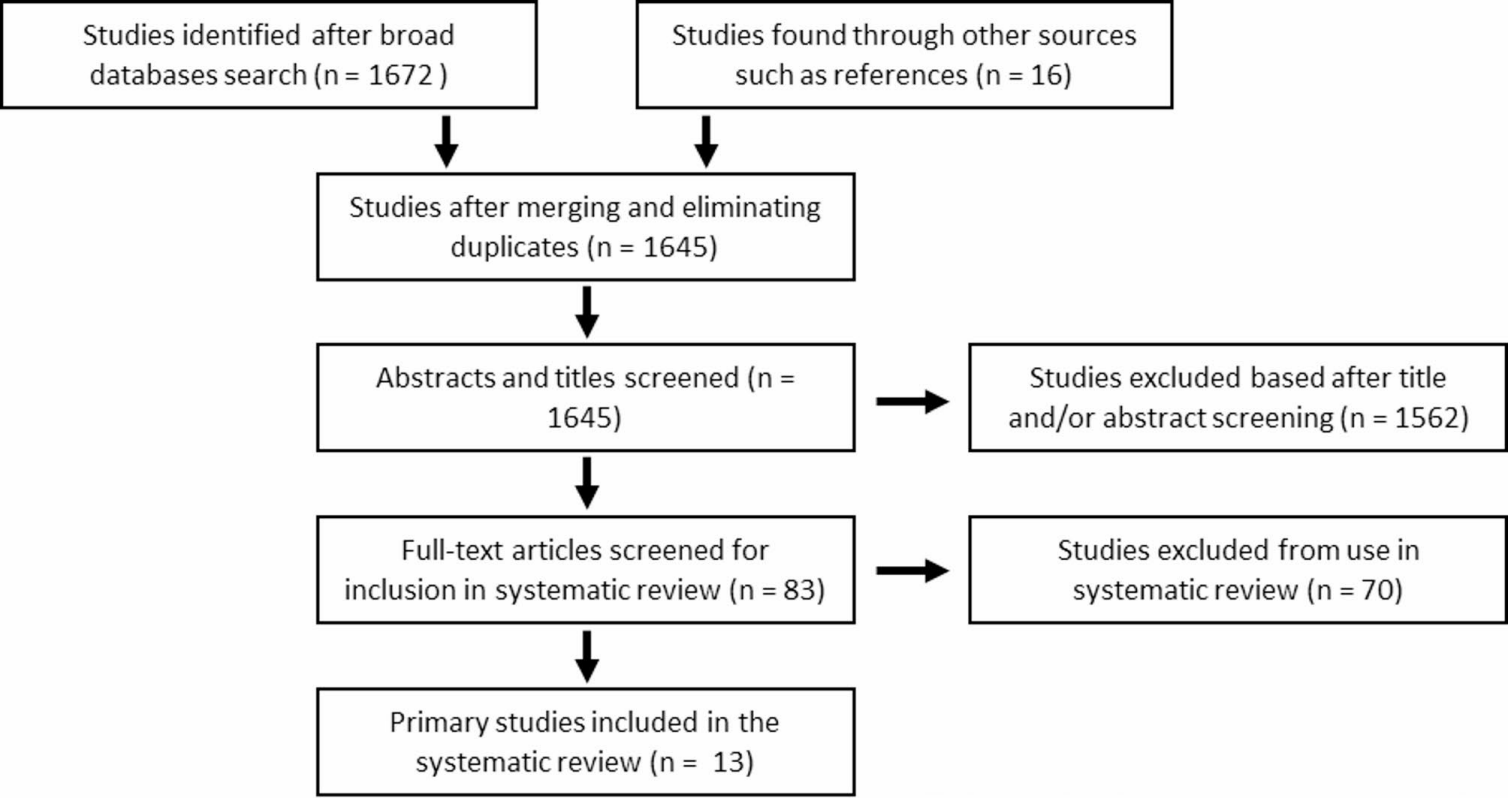
PRISMA flowchart diaphragm from literature search to final analysis.

**Fig. 2:**
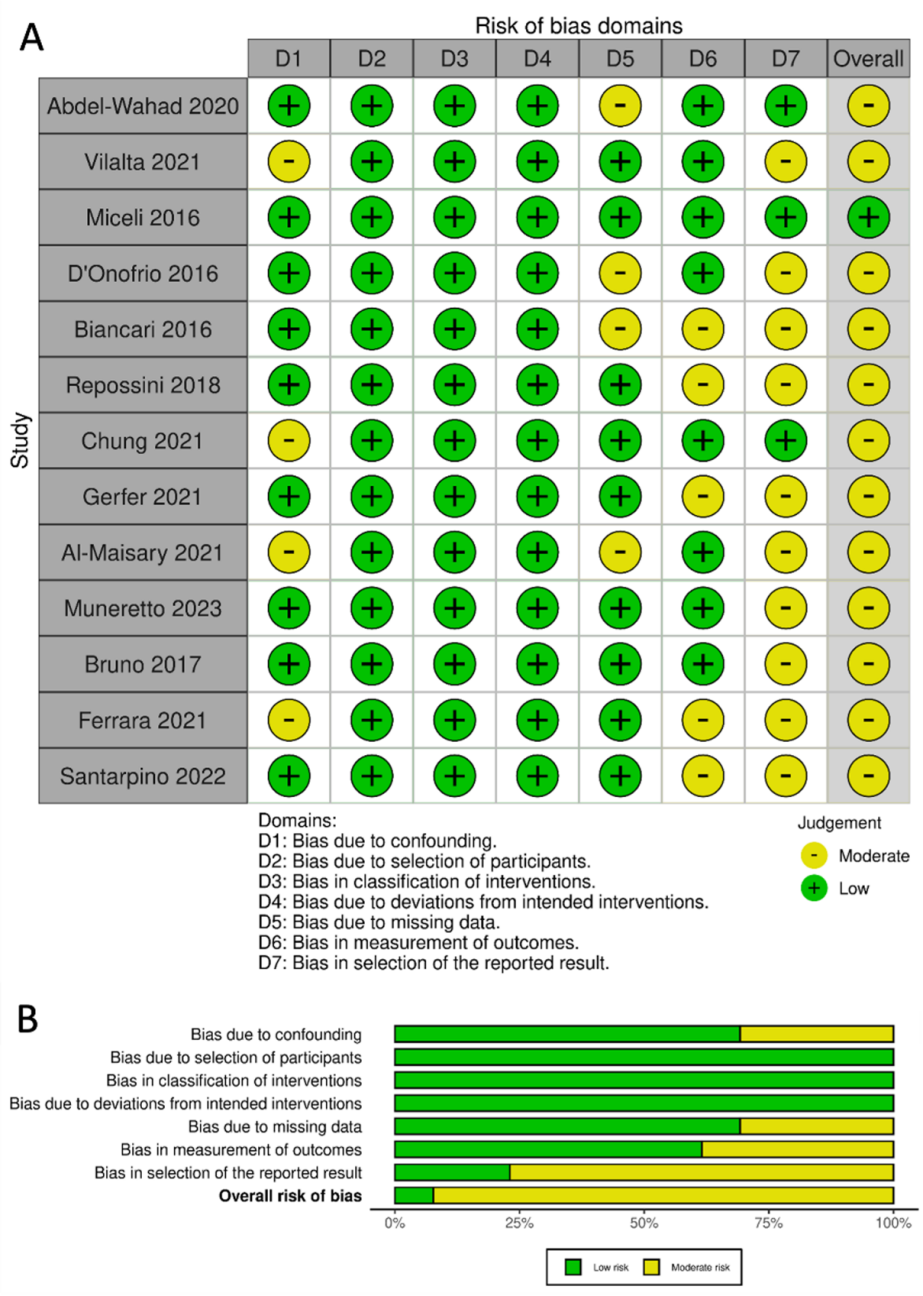
ROBINS-I-tool risk of bias summary with (**A**) traffic light plot and (**B**) summary plot. ROBINS-I: Risk of bias in non-randomized studies of interventions.

Studies that were included in this review directly compared TAVR with SU-AVR, using a propensity score matched analysis. Abstracts, expert opinions and editorials, case reports and case series with less than ten patients were excluded. If more studies were published from the same author, center or database, only the one with the most complete dataset was included. There were no restrictions on publication language, but all studies used in the analysis were in English. An overview of the studies is presented in (Fig. 1). Three studies were manually excluded: one very small non-propensity matched study^8^; another study by Kamperidis et al. because of the use of the 3f Enable sutureless valve, which was taken off the market in 2015^9^. Lastly, a low-risk study by Muneretto et al. was excluded for risk of overlap with another study from the same author in patients at intermediate risk, the latter study being included^10^. The quality of studies was assessed by determining the level of evidence according to the criteria provided by the Oxford Centre for Evidence-based Medicine. All thirteen studies were retrospective matched cohort studies giving them a level 3b evidence. Every study has limitations besides the low number of patients, the absence of prospective randomization and propensity matching, not fully accounting for selection bias. Risk of bias was investigated using the ROBINS-I-tool and is shown in (Fig. 2).

### Study outcomes

The main study outcomes are in-hospital, 30-day, and 1 and 2-year survival. Other frequently reported outcomes are postoperative complications including stroke, acute kidney injury (AKI) and permanent pacemaker implantation (PPI). With regards to hemodynamics, most studies present postoperative echocardiographic parameters such as more than mild (para)valvular leakage (PVL) and the mean and peak transprosthetic gradients (TPG). Some studies also include these parameters in follow-up evaluations. Interestingly, some studies also report length of stay in the intensive care unit (ICU) or hospital, sometimes including a cost analysis of different techniques.

### Statistical analysis

Meta-analysis was performed using SAS software (version 9.4) on the log Odds Ratios (OR) of binary outcomes (in-hospital mortality, 30-day mortality, stroke, PPI, AKI, PVL ≥ grade 2), on the log Hazard Ratio (HR) of the all-cause mortality survival outcome and on continuous outcomes (peak and mean postoperative TPG). For the continuous outcomes, medians and quartiles were transformed into means and standard deviations following Luo et al. (2017), and Wan et al. (2014)^11,12^. A sensitivity analysis was performed that excludes this transformed information to examine the robustness of the results.

The random-effects approach of DerSimonian and Laird^13^ was used to obtain a combined estimate. Between-study heterogeneity was evaluated using Chi² and I² statistics: I² = 100%*(Chi² - df)/df, where df is the degrees of freedom corresponding to the Chi² statistic^14^. I^2^ is the percentage of total variation in study estimates that is due to heterogeneity. Prediction intervals were reported alongside the effect estimates to show the extent of between-study variation. These analyses were performed overall and per surgical-risk subgroup. The results of the meta-analysis are presented using forest plots displaying the effect estimates and confidence intervals for the individual studies, the risk subgroups and the pooled study population. Funnel plots (Supplemental) were used to assess the publication bias of the included studies, with the addition of Egger’s regression tests. For none of the funnel plots was the p-value of the Egger’s regression test smaller than 0.05, indicating that the studies were symmetrically distributed. However, caution is warranted as the Egger’s regression test may be underpowered when the number of studies is lower than 10, which is the case for acute kidney injury, peak and mean postoperative gradient and all-cause mortality.

To perform a meta-analysis on the survival data and estimate the underlying Individual Person Data (IDP) from the Kaplan-Meier (KM) curves, the package IPDfromKM was used in Rstudio (version 2023.9.1.494)^15^. Then two statistical strategies were utilized. Firstly, we gathered the hazard ratios that were available in some individual studies and calculated their standard errors based on the confidence intervals. We combined them with hazards ratios and standard errors obtained from Cox regressions on the IPD data for which only Kaplan-Meier curves were available. Then we performed a random-effects meta-analysis on the log(HR). Secondly, we combined the IPD data from the individual studies into two Kaplan-Meier curves, one for SU-AVR and one for TAVR, and the 1-year and 2-year survival probabilities were estimated with 95% confidence intervals (CI). The two resulting Kaplan-Meier curves were compared with the log-rank test.

Weighted averages for each risk group were calculated as the sum of (number/percentage reported by a study multiplied by the number of patients in the study) for each study and then divided by the total patient number for each risk group.

## Results

### Patient characteristics

Baseline patient characteristics were similar between the two treatment groups due to propensity score matching across all studies (Table 1). Mean age ranged from 75.0 to 82.6 years. Surgical risk scores were reported using the logistic EuroSCORE, EuroSCORE II or STS score. Studies were stratified according to surgical risk, with a low (< 10%; < 1.5%; <4%), intermediate (10–20%; 1.6-5.0%; 4–8%) and high-risk group (> 20%; > 5%; > 8%), respectively^16–18^. The risk categorization of the study from Chung et al. 2021 was assumed to be intermediate based on the reported number of patients with a EuroSCORE II of ≥ 4%.

**Table 1:** Patient characteristics.

	Abdel-Wahab et al. (2020)^19^	Vilalta et al. (2021)^20^	Miceli et al. (2016)^24^	D’Onofrio et al. (2016)^25^	Biancari et al. (2016)^21^	Repossini et al. (2018)^22^	Chung et al. (2021)^26^	Gerfer et al. (2021)^27^	Al-Maisary (2021)^23^	Muneretto et al. (2023)^28^	Bruno et al. (2017)^29^	Ferrara et al. (2021)^31^	Santarpino et al. (2022)^30^
Valve types and nr of patients	Perceval = 972 Intuity = 633 TAVR = 1605	Perceval = 171 TAVR = 171	Perceval = 37 TAVR = 37	Perceval = 214 TAVR = 214	Perceval = 144 TAVR = 144	Perceval = 185 TAVR = 185	Perceval = 60 Intuity = 2 TAVR = 62	Perceval = 59 TAVR = 59	Perceval = 35 Intuity = 16 TAVR = 52	Perceval = 517 TAVR = 517	Intuity = 30 TAVR = 30	Intuity = 48 TAVR = 48	Perceval = 172 TAVR = 172
Surgical risk	Low	Low	Intermediate	Intermediate	Intermediate	Intermediate	Intermediate	Intermediate	Intermediate	Intermediate	High	High	High
Age (years)	75 (70–78) vs. 78 (73–81) (*P* < .001)	78.0 ± 5.7 vs. 77.4 ± 8.4 (*P* = .384)	79 ± 4.5 vs. 78.8 ± 7.4 (*P* = .92)	77.4 ± 5.4 vs. 77.7 ± 7.9 (*P* = .07)	79.4 ± 5.4 vs. 79.0 ± 6.0 (*P* = .745)	76 ± 4 vs. 76 ± 4	75.5 ± 5.3 vs. 76.8 ± 6.0 (*P* = .203)	77 ± 8 vs. 79 ± 5	75 ± 4 vs. 77 ± 4.3 (*P* = .051)	81 (78–84) vs. 82 (77.2–85) (*P* = .139)	79.9 ± 3.6 vs. 81.1 ± 3.3 (*P* = .2)	79.5 ± 6.0 vs. 82.6 ± 5.8 (*P* = .01)	80.9 ± 5.1 vs. 79.1 ± 7.4 (*P* = .1)
Male	670 (41.7) vs. 637 (39.7) (*P* = .24)	64 (37.4) vs. 62 (36.3) (*P* = .823)	13 (35.1) vs. 15 (40.5)	76 (35.5) vs. 75 (35.0)	56 (38.2) vs. 54 (37.5)	118 (63.8) vs. 119 (64.3)	24 (38.7) vs. 20 (32.3) (*P* = .453)	21 (36) vs. 21 (36)	20 (30) vs. 20 (30)	194 (37.5) vs. 198 (38.3) (*P* = .797)	15 (50) vs. 17 (56.7)	27 (56.3) vs. 24 (50.0) (*P* = .54)	67 (39) vs. 74 (43.1)
BMI (kg/m^2^)	27.8 (24.9–31.3) vs. 27.4 (24.5–31.1) (*P* = .1)	29.3 ± 5.0 vs. 29.2 ± 7.2 (*P* = .912)	NR	27.5 ± 4.7 vs. 27.6 ± 5.2 (*P* = .98)	NR	27.1 ± 5.1 vs. 27.5 ± 5.7	24.9 ± 3.3 vs. 24.9 ± 3.4 (*P* = .986)	28 ± 6 vs. 28 ± 6	28 ± 5 vs. 27.3 ± 5 (*P* = .440)	26 (23.8–28.5) vs. 26 (23.2–29.5) (*P* = .855)	25.8 ± 2.7 vs. 26.4 ± 2.4 (*P* = .37)	24.9 ± 3.8 vs. 26.1 ± 4.7 (*P* = .19)	26.3 ± 2.9 vs. 26.7 ± 3.4 (*P* = .2)
Diabetes	152 (9.5) vs. 180 (11.2) (*P* = .11)	52 (30.4) vs. 59 (34.5) (*P* = .419)	10 (27) vs. 7 (18.9) (*P* = .62)	59 (27.6) vs. 58 (27.1) (*P* = .91)	6 (4.2) vs. 5 (3.5) (*P* = .759)	46 (24.9) vs. 48 (25.9)	24 (38.7) vs. 23 (37.1) (*P* = .853)	18 (31) vs. 16 (27)	22 (42) vs. 21 (40) (*P* = .971)	160 (30.9) vs. 165 (31.9) (*P* = .738)	6 (20) vs. 9 (31) (*P* = .34)	9 (18.8) vs. 15 (31.3) (*P* = .16)	24 (13.9) vs. 32 (18.6) (*P* = .3)
Previous cardiac surgery	80 (5.0) vs. 67 (4.2) (*P* = .27)	7 (4.1) vs. 10 (5.9) (*P* = .455)	3 (8.1) vs. 3 (8.1) (*P* = 1)	22 (10.3) vs. 21 (9.8) (*P* = .87)	12 (8.3) vs. 15 (10.4) (*P* = .544)	13 (7.0) vs. 15 (8.1)	5 (8.1) vs. 4 (6.5) (*P* > .999)	2 (3) vs. 3 (5)	NR	43 (8.3) vs. 45 (8.7) (*P* = .824)	NR	6 (12.5) vs. 5 (10.4) (*P* = .74)	15 (8.7) vs. 26 (15.1) (*P* = .1)
PAD	68 (4.2) vs. 68 (4.2) (*P* = 1)	14 (8.2) vs. 12 (7.0) (*P* = .683)	11 (29.7) vs. 9 (24.3) (*P* = .79)	46 (21.5) vs. 48 (22.4) (*P* = .81)	12 (8.3) vs. 13 (9.0) (*P* = .834)	32 (17.3) vs. 36 (19.5)	4 (6.5) vs. 7 (11.3) (*P* = .343)	10 (17) vs. 11 (19)	8 (15) vs. 15 (29) (*P* = .98)	125 (24.2) vs. 129 (24.9) (*P* = .773)	12 (40) vs. 9 (30) (*P* = .59)	NR	22 (12.7) vs. 31 (18) (*P* = .3)
LVEF (%)	NR	60.0 ± 12.6 vs. 60.4 ± 5.6 (*P* = .695)	52.6 ± 9.7 vs. 50.6 ± 7.8 (*P* = .5)	57.7 ± 9.3 vs. 58.2 ± 10.2 (*P* = .41)	NR	55.1 ± 7.3 vs. 55.4 ± 6.8	60.8 ± 13.8 vs. 62.2 ± 14.6 (*P* = .605)	NR	NR	60 (50–63) vs. 60 (50–65) (*P* = .644)	NR	58.8 ± 10.5 vs. 56.6 ± 13.5 (*P* = .39)	48.6 ± 7.2 vs. 49.6 ± 9.9 (*P* = .2)
Logistic EuroSCORE	6.2 (4.5–9.2) vs. 7.5 (5.5–10.9) (*P* < .001)	NR	16.1 ± 11 vs. 15.7 ± 8.5 (*P* < .0001)	10.5 ± 6.2 vs. 12.4 ± 9.1 (*P* = .09)	NR	NR	NR	NR	17 ± 10 vs. 19 ± 12 (*P* = .257)	NR	NR	NR	NR
EuroSCORE II (%)	NR	1.9 (1.3–2.5) vs. 1.9 (1.3–2.5) (*P* = .851)	NR	NR	4.1 ± 3.2 vs. 3.6 ± 2.6 (*P* = .117)	NR	NR	2.5 ± 1.2 vs. 2.5 ± 1.2	NR	6.0 (4–6.63) vs. 5.5 (4.3–7.5) (*P* = .67)	5.0 ± 0.9 vs. 5.2 ± 1.2 (*P* = .5)	6.6 ± 1.8 vs. 5.6 ± 1.5 (*P* = .005)	5.6 ± 2.9 vs. 6.1 ± 1.5 (*P* = .3)
STS-PROM, %	2.2 vs. 2.7 (*P* < .001)	2.8 vs. 2.6 (*P* = .901)	NR	NR	NR	5.2 vs. 5.3	NR	NR	3.9 vs. 4.5 (*P* = .339)	6.0 (4–6) vs. 5.9 (4.0-7.1) (*P* = .289	NR	NR	NR

Data are expressed as mean ± standard deviation, median (interquartile range) or n (%), Sutureless/rapid-deployment vs. TAVR.

SAVR: surgical aortic valve replacement; SU-AVR: Sutureless aortic valve replacement; TAVR: transcatheter aortic valve replacement; BMI: body mass index; PAD: peripheral artery disease; LVEF: left ventricle ejection fraction; EuroSCORE: European System for Cardiac Operative Risk Evaluation; NR: not reported.

### Overall

**Fig. 3:**
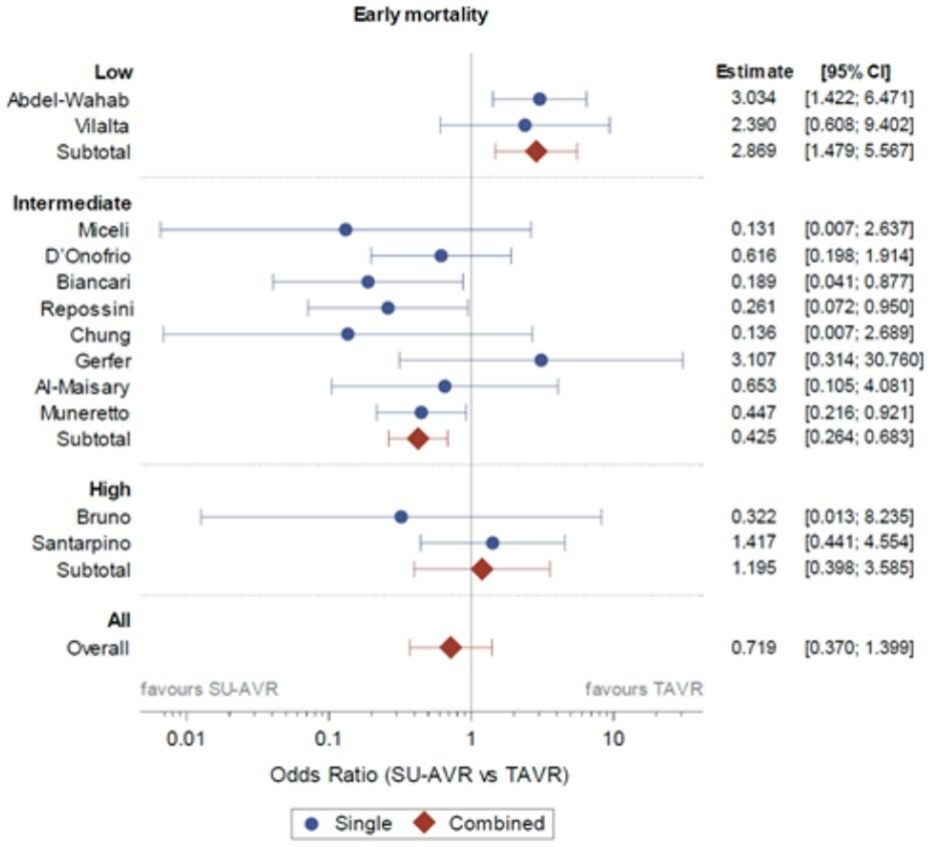
Forest-plot of early mortality showing Odds ratio and 95% confidence interval. SU-AVR: sutureless aortic valve replacement, TAVR: transcatheter aortic valve replacement.

**Fig. 4:**
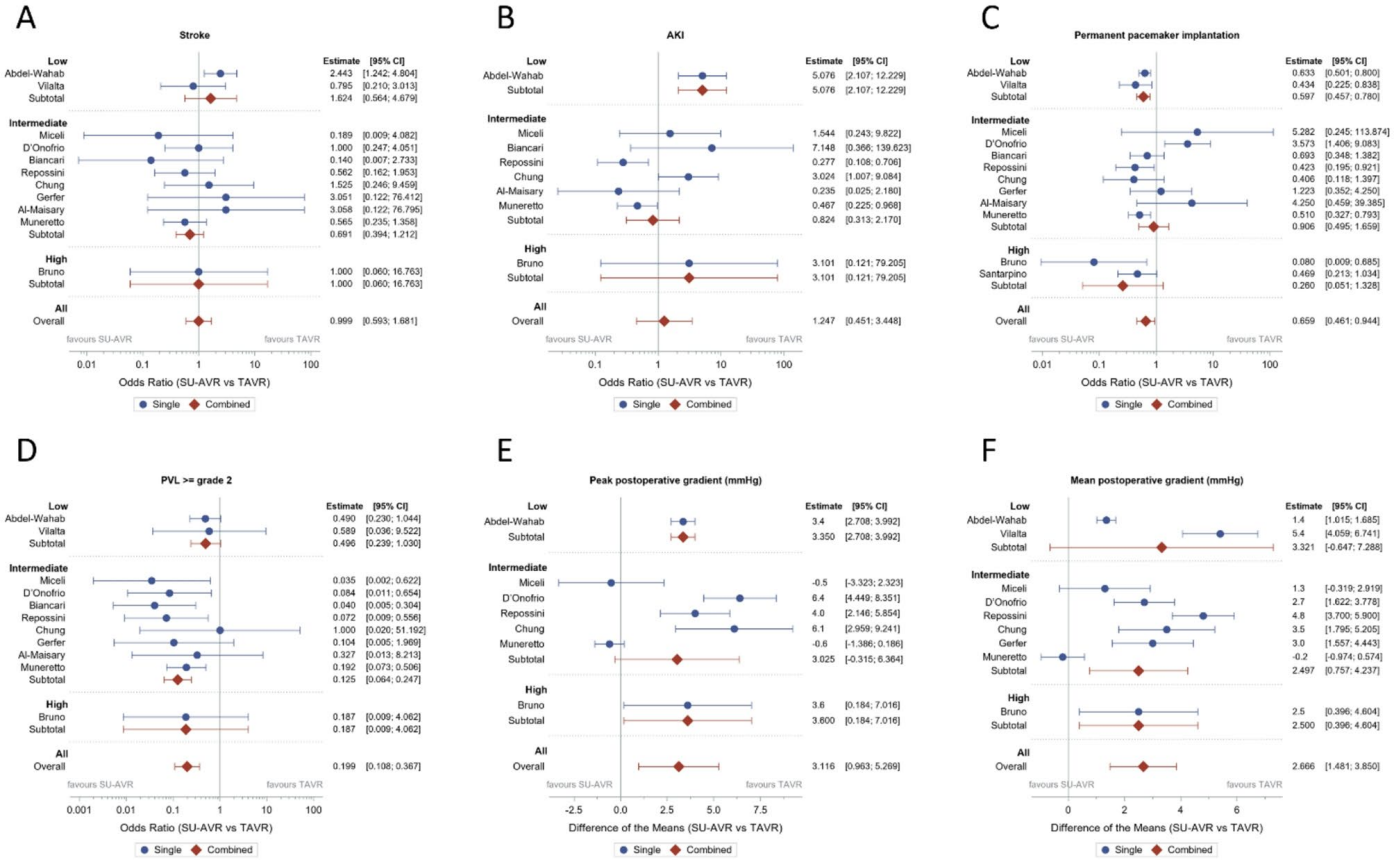
Forest-plots of postoperative complication rates and hemodynamic performance showing Odds ratio or difference of the mean and 95% confidence interval. (**A**) Stroke; (**B**) AKI: acute kidney injury; (**C**) Permanent pacemaker implantation; (**D**) PVL: paravalvular leakage; (**E**) Peak postoperative gradient, (**F**) Mean postoperative gradient. SU-AVR: sutureless aortic valve replacement, TAVR: transcatheter aortic valve replacement.

**Fig. 5:**
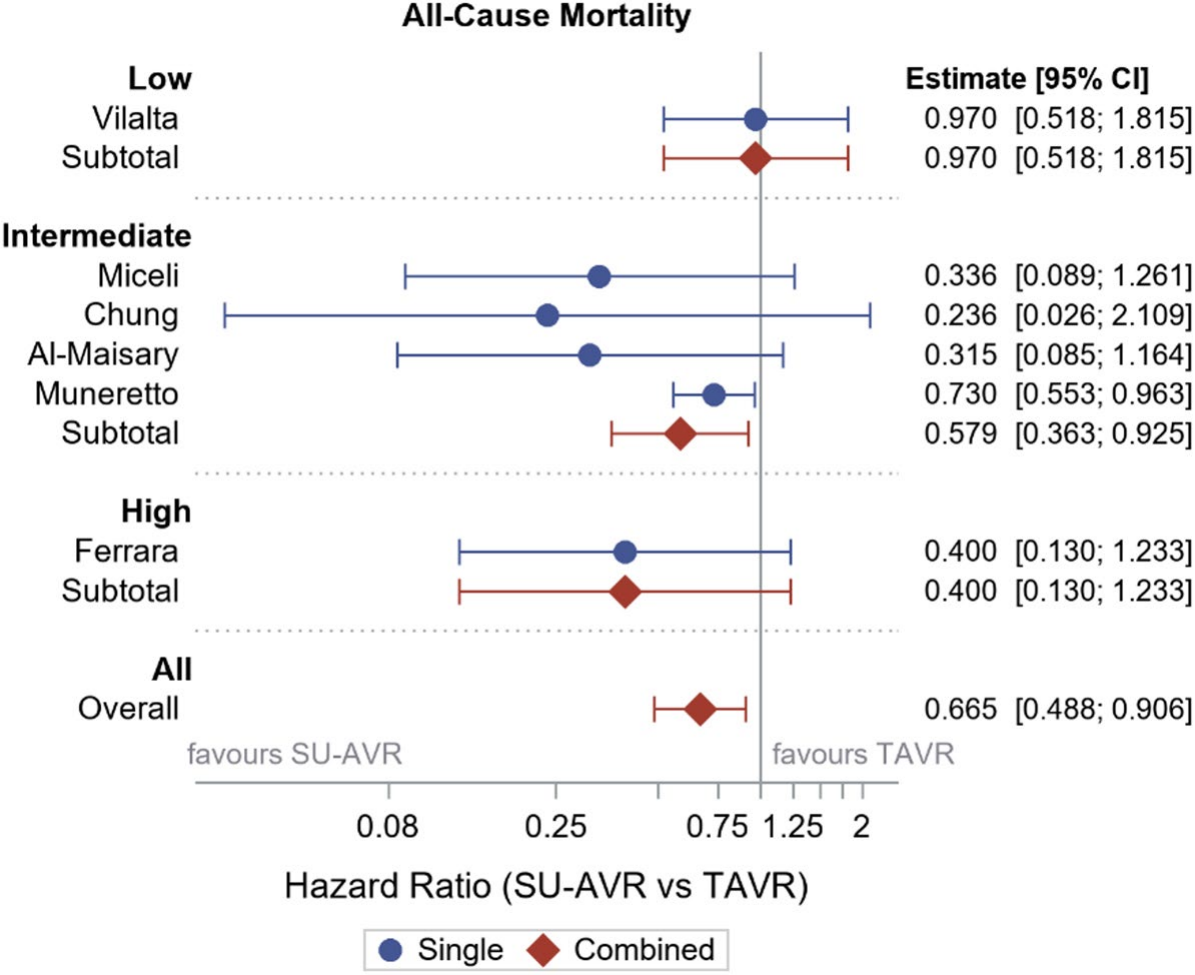
Forest plot showing Hazard Ratios and 95% confidence intervals of all-cause mortality. SU-AVR: sutureless aortic valve replacement, TAVR: transcatheter aortic valve replacement.

**Fig. 6:**
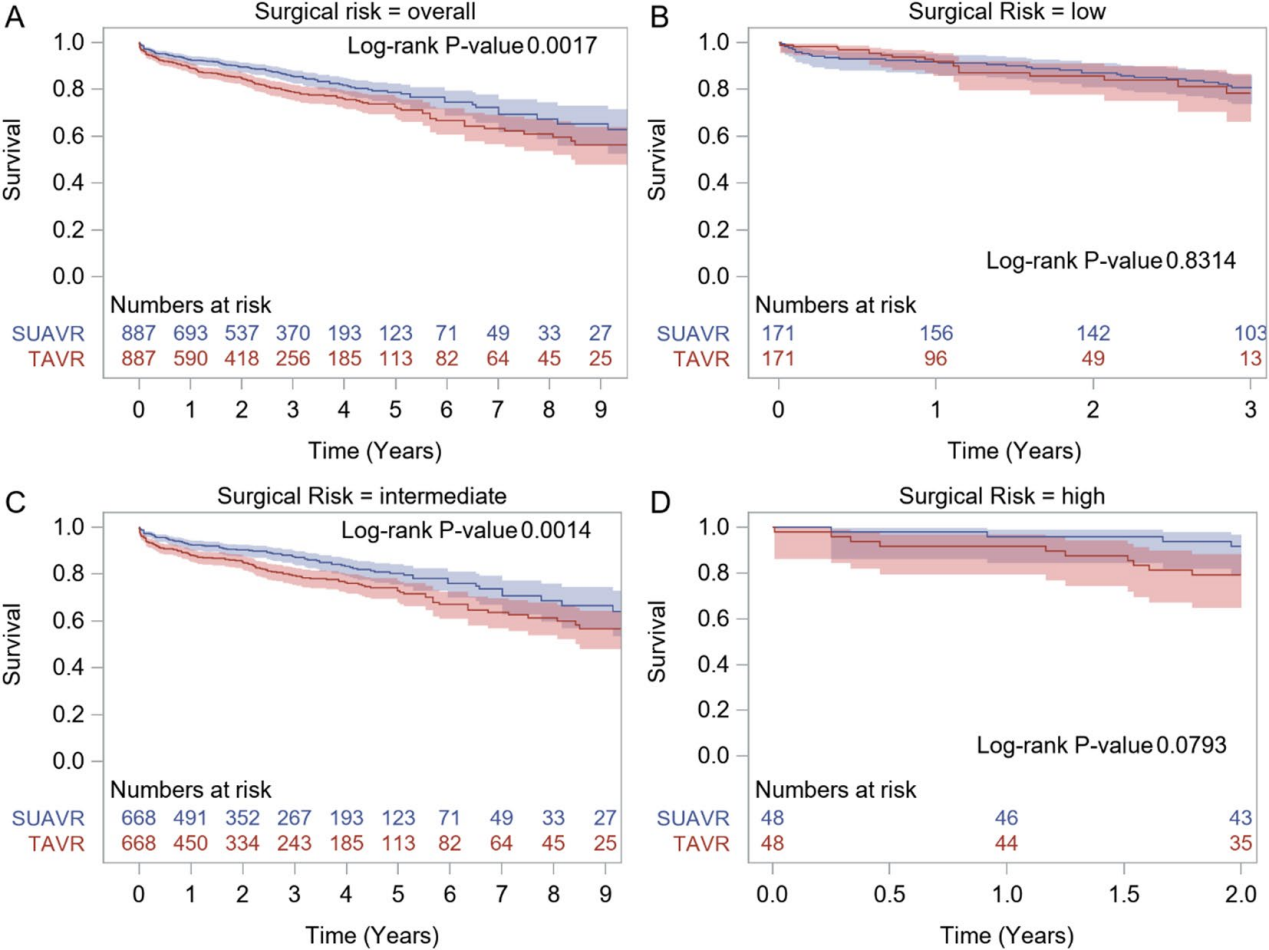
Kaplan-Meier plots showing pooled overall survival. (**A**) Overall; (**B**) Low-risk patients; (**C**) Intermediate-risk patients; (**D**) High risk patients. SU-AVR: sutureless aortic valve replacement, TAVR: transcatheter aortic valve replacement.

When considering the entire patient population there was no difference in early mortality (combining 30 days and in-hospital mortality) between SU-AVR and TAVR (OR 0.719 (95% CI: 0.370–1.399) (Fig. 3). No difference in stroke (OR 0.999 (95% CI: 0.593–1.681)) or AKI (OR 1.247 (95% CI: 0.451–3.448) was seen (Fig. 4A-B). SU-AVR was associated with less permanent pacemaker implantation (OR 0.659), less PVL (graded at least moderate) (OR 0.199), but slightly higher post-operative gradients (peak gradient: 3.108 mmHg (CI: 0.136–6.079)) and higher mean gradients: 2.866 mmHg (CI: 1.292–4.439))(Fig. 4C-F). Overall, in these observational studies, SU-AVR seems to be related with a lower mortality rate (HR: 0.665 CI: 0.488, 0.906)(Fig. 5) and the Kaplan-Meier curves are significantly different, favouring SU-AVR (1-year mortality: SU-AVR: 6.23% (CI: 4.9–7.9%) vs. TAVR: 9.15 (CI: 7.5–11.1%), 2-year mortality: SU-AVR: 9.90% (CI 8.1–12.1%) vs. TAVR: 13.87% (CI: 11.7–16.4%), Log-rank *p* = .0017)(Fig. 6A).

**Fig. 7:**
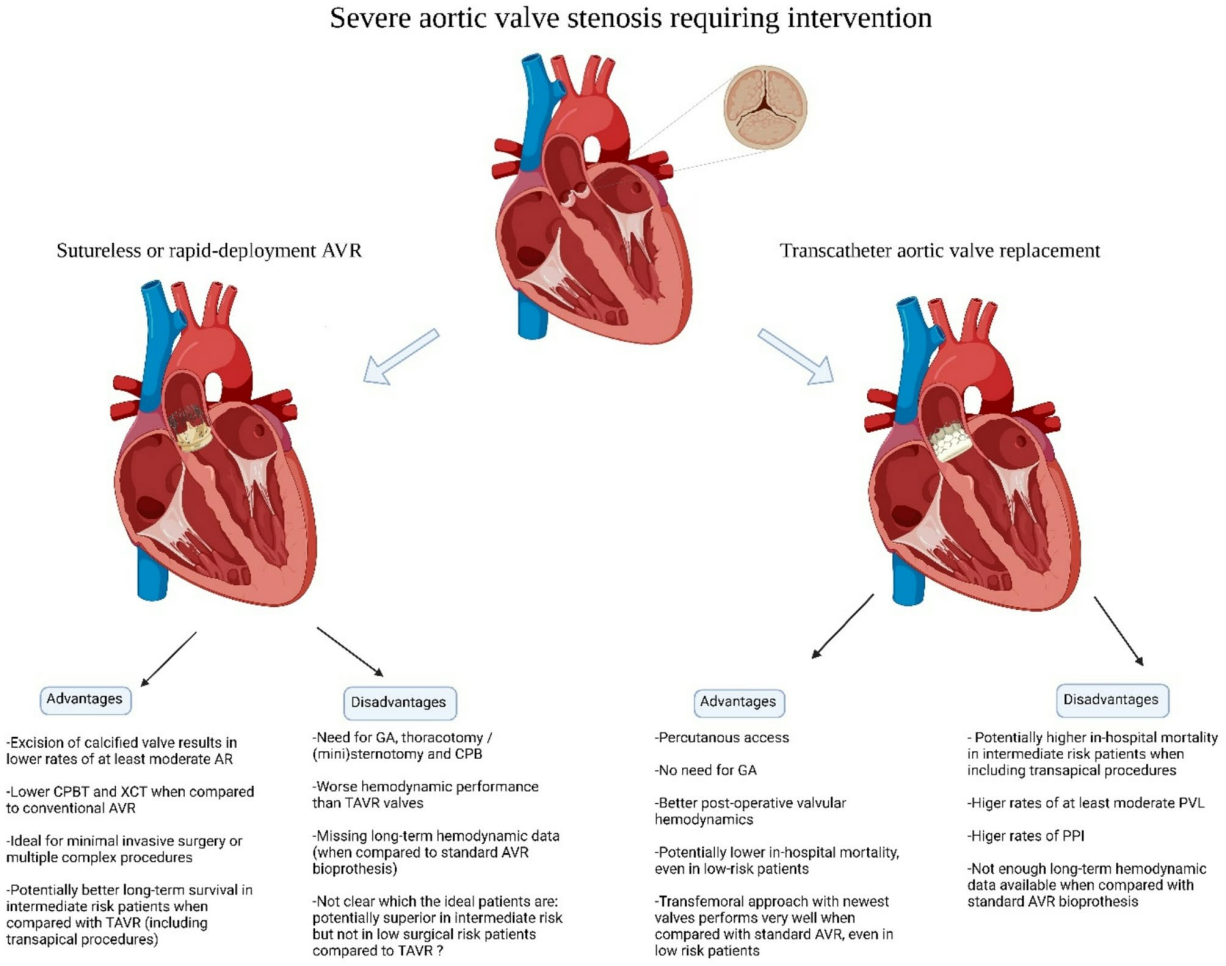
Summary of upsides and downsides of SU-AVR and TAVR. AVR: aortic valve replacement, CPB(T): cardio-pulmonary bypass (time), GA: general anesthesia, PPI: permanent pacemaker implantation, PVL: paravalvular leakage, SU-AVR: sutureless aortic valve replacement, TAVR: transcatheter aortic valve replacement, XCT: aortic cross-clamp time.

### Low-risk patients

#### Primary endpoint

Only two studies included low risk patients, both reporting early mortality (OR: 2.869 with 95% CI 1.479–5.567) (Fig. 3), which was significantly higher for SU-AVR in the study of Abdel-Wahad et al. (Table 2)^19,20^. This report comes from the German aortic valve registry (GARY) and includes two types of surgical valves (the sutureless Perceval and the rapid-deployment Intuity) (Fig. 7). Vilalta et al. found similar mortality and stroke rates, but with an increased risk of heart failure hospitalization for SU-SAVR patients at 2-year follow-up (Table 2). No significant difference for all-cause mortality was observed between both procedures (1-year mortality: SU-AVR: 8.19% vs. TAVR: 8.13%, 2-year mortality: SU-AVR: 13.02% vs. TAVR: 14.28%, HR: 0.97 (CI: 0.52, 1.82), *p* = .936) (Figs. 5 and 6B).

**Table 2:** Outcome.

	Abdel-Wahab et al. (2020) ^19^	Vilalta et al. (2021) ^20^	Miceli et al. (2016) ^24^	D’Onofrio et al. (2016) ^25^	Biancari et al. (2016) ^21^	Repossini et al. (2018) ^22^	Chung et al. (2021) ^26^	Gerfer et al. (2021) ^27^	Al-Maisary (2021) ^23^	Muneretto et al. (2023) ^28^	Bruno et al. (2017) ^29^	Ferrara et al. (2021) ^31^	Santarpino et al. (2022) ^30^
Valve types and nr of patients	Perceval = 972 Intuity = 633 TAVR = 1605	Perceval = 171 TAVR = 171	Perceval = 37 TAVR = 37	Perceval = 214 TAVR = 214	Perceval = 144 TAVR = 144	Perceval = 185 TAVR = 185	Perceval = 60 Intuity = 2 TAVR = 62	Perceval = 59 TAVR = 59	Perceval = 35 Intuity = 16 TAVR = 52	Perceval = 517 TAVR = 517	Intuity = 30 TAVR = 30	Intuity = 48 TAVR = 48	Perceval = 172 TAVR = 172
Surgical risk	Low	Low	Intermediate	Intermediate	Intermediate	Intermediate	Intermediate	Intermediate	Intermediate	Intermediate	High	High	High
Early mortality (%)	1.7% vs. 0.6% (*P* = .003)	4.1% vs. 1.8% (*P* = .199)	0% vs. 8.1% (*P* = .25)	2.3% vs. 3.7% (*P* = .39)	1.4% vs. 6.9% (*P* = .035)	1.6% vs. 5.9%	0% vs. 4.8% (*P* = .244)	5.1% vs. 1.7% (*P* = .619)	3.8% vs. 5.8% (*P* = .647)	2.1% vs. 4.6% (*P* = .026)	0% vs. 3.3% (*P* = .330)	NR	4% vs. 2.9% (*P* = .7)
1-year mortality (%)	NR	NR	8.4% vs. 21.4%*	5.8% vs. 9.4% (*P* = .16)	NR	NR	1.7% vs. 7.0% (*P* = .149)	NR	6% vs. 27% (*P* = .058) *	NR	NR	4.2% vs. 8.3% *	NR
2-year mortality (%)	NR	18.1% vs. 9.9% (*P* = .936)	8.4% vs. 33.8%*	NR	NR	4.9% vs. 10.9%*	NR	NR	NR	NR	NR	8.4% vs. 20.8% *	NR
Stroke (%)	1.8% vs. 0.7% (*P* = .01)	2.3% vs. 2.9% (*P* = .736)	0% vs. 5.4% (*P* = .3)	1.9% vs. 1.9% (*P* > .99)	0% vs. 2.1% (*P* = .122)	Stroke or TIA 2.1% vs. 3.7%	4.8% vs. 3.2% (*P* = 1)	1.7% vs. 0.0% (*P* = 1)	1.9% vs. 0% (*P* = .315)	Stroke or TIA 1.5% vs. 2.7% (*P* = .596)	3.3% vs. 3.3% (*P* = .980)	NR	NR
AKI (%)	1.9% vs. 0.4% (< 0.001)	NR	8.1% vs. 5.4% (*P* = .45)	NR	2.1% vs. 0% (*P* = .247)	3.2% vs. 10.8%	21.0% vs. 8.1% (*P* = .041)	NR	1.9% vs. 7.7% (*P* = .169)	2.1% vs. 4.4% (*P* = .036)	3.3% vs. 0% (*P* = .33)	NR	NR
Permanent pacemaker implantation (%)	8.5% vs. 12.8% (*P* < .001)	8.8% vs. 18.1% (*P* = .011)	5.4% vs. 0.0% (*P* = .5)	9.4% vs. 2.8% (*P* = .0046)	11.2% vs. 15.4% (*P* = .296)	5.4% vs. 11.9%	6.5% vs. 14.5% (*P* = .143)	10.2% vs. 8.5% (*P* = .752)	7.7% vs. 1.9% (*P* = .169)	6.4% vs. 11.8% (*P* = .002)	3.3% vs. 30% (*P* = .01)	-	5.8% vs. 11.6% (*P* = .1)
ICU LOS (days)	2 (1–4) vs. 1 (1–2) (*P* < .001)	NR	1 (1–2) vs. 1 (1–1) (*P* = .5)	2 (1–3) vs. 1 (1–3) (*P* < .001)	NR	1.6 ± 2.8 vs. 3.2 ± 2	5.9 ± 9.2 vs. 1.9 ± 1.6 (*P* = .009)	4 ± 5 vs. 3 ± 3 (*P* = .279)	NR	1 (0-1.5) vs. 1 (0–2) (*P* = .258)	3.1 ± 1.4 vs. 2.1 ± 1.0 (*P* = .001)	NR	NR
Hospital LOS (days)	10 (8–14) vs. 7 (5–9) (*P* < .001)	NR	7 (6–8) vs. 4.5 (3–6) (*P* < .001)	11 (8–16) vs. 7 (5–9) (*P* < .001)	NR	8.1 ± 11.3 vs. 9.5 ± 6.5	13.1 ± 8.8 vs. 7.1 ± 7.9 (*P* < .001)	12 ± 5 vs. 9 ± 5 (*P* = .001)	NR	NR	7.7 ± 2.9 vs. 9.4 ± 2.6 (*P* = .02)	-	12 vs. 8 (*P* < .01)
Peak postoperative gradient (mmHg)	23 (17–30) vs. 20 (14–26) (*P* < .001)	NR	19.2 ± 6.9 vs. 19.7 ± 5.4 (*P* = .26)	26.7 ± 12.1 vs. 20.3 ± 8.1 (*P* < .001)	NR	18.7 ± 9.1 vs. 14.7 ± 9.1	27.5 ± 7.0 vs. 21.4 ± 10.5 (*P* < .001)	NR	NR	21.8 ± 6.5 vs. 22.4 ± 6.4 (*P* = .424)	20.3 ± 6.7 vs. 16.7 ± 6.8 (*P* = .04)	NR	NR
Mean postoperative gradient (mmHg)	12 (9–16) vs. 11 (8–14) (*P* < .001)	16.8 ± 6.9 vs. 11.4 ± 5.7 (*P* < .001)	11.4 ± 3.7 vs. 10.1 ± 3.4 (*P* = .17)	13.7 ± 6.6 vs. 11.0 ± 4.6 (*P* < .001)	NR	10.9 ± 5.4 vs. 6.1 ± 5.4	14.7 ± 3.8 vs. 11.2 ± 5.7 (*P* < .001)	12 ± 4 vs. 9 ± 4 (*P* = .001)	NR	10.6 ± 6.2 vs. 10.8 ± 6.5 (*P* = .786)	11.5 ± 3.9 vs. 9.0 ± 4.4 (*P* = .03)	NR	NR
PVL ≥ grade 2	0.7% vs. 1.3% (*P* = .06)	0.6% vs. 1.1% (*P* = .706)	0% vs. 27%	0.5% vs. 5.3%	0.7% vs. 14.8%	0.5% vs. 7.0%	0% vs. 0%	0% vs. 6.8% (*P* = .119)	0% vs. 1.9%	0.97% vs. 4.8% (*P* < .001)	0% vs. 6.6% (*P* = .33)	NR	NR
TAVR valve types	Sapien 3: 1065 Evolut: 549	Sapien 3: 99 Evolut PRO: 34 Portico: 25 Acurate NEO: 8 Direct flow: 5	Sapien	Sapien; Sapien XT	CoreValve: 286 Sapien XT: 99 Portico: 2 Lotus: 2	CoreValve: 238 Sapien XT: 117 Acurate TA: 12	CoreValve: 16 Evolut R: 25 Evolut Pro: 3 Sapien 3: 15 Lotus: 3	Acurate NEO	JenaValves: 5 Evolut R: 1 Sapien XT: 18 Sapien 3: 22	Evolut R/pro Acurate TA Acurate neo/neo2 Sapien XT/3	Corevalve	Sapien 3	CoreValve
TAVR approach	TF: 100%	TF: 77.2% Tcar: 18.1% Tax 3.5% Tao 1.2%	TF: 51.6% TA: 48.3% unmatched	NR	TF: 97.7% TA: 1.0% Tsub: 0.8% Tao: 0.5% unmatched	TF: 72.8% TA: 22.4% Other: 4.8%	TF: 98% Tsub: 1.2% Tao: 0.8%	TF: 100%	TA: 100%	TF: 79.4% TA: 15.2% Other: 5.3%	TF: 100%	TF: 81.7% Tcar: 1.9% Tsub: 5.8% Tao: 7.7% TA: 2.9%	TF: 100%

Data are expressed as mean ± standard deviation, median (interquartile range) or n (%), Sutureless/rapid-deployment vs. TAVR.

SAVR: surgical aortic valve replacement; SU-AVR: Sutureless aortic valve replacement; TAVR: transcatheter aortic valve replacement; TF: transfemoral; TA: transapical; Tao: Transaortic; Tsub: transsubclavian; Tax: transaxillary: Tcar: transcarotid; NR: not reported; ICU: intensive care unit; LOS: length of stay, NR: not reported, AR: aortic regurgitation.

* in 1- or 2-year mortality is derived from Kaplan-Meyer estimates.

#### Secondary endpoints

Postoperative PPI was significantly lower in SU-AVR for both studies (OR: 0.597, CI 0.457–0.780), which was confirmed in our meta-analysis (Fig. 4C). Stroke rate was found to be significantly lower for TAVR by Abdel-Wahab et al. (OR: 2.442, CI: 1.242–4.804);^19,20^. Nevertheless, our meta-analysis could not show a significant difference between the two procedures regarding stroke in low-risk patients (Fig. 4A). The GARY registry also reported AKI (OR: 5.076 with CI: 2.107–12.229) (Fig. 4B), ICU stay (sutureless/RDV: 2 days vs. TAVR: 1 day, *p* < .001) and hospital length of stay (sutureless/RDV: 10 days vs. TAVR: 7 days, *p* < .001)^19^. The results of these outcomes are presented in Fig. 4; Table 2.

#### Hemodynamic performance at discharge

Hemodynamic performance was reported in both studies, with significantly lower mean TPG for TAVR. Meta-analysis: Difference of the means (DoM): 3.321 with 95% CI (-0.647–7.288). More than mild PVL (OR: 0.496 with CI (0.239–1.030) showed a non-significant trend towards less PVL in the low-risk SU-AVR patients. (Fig. 4D)^19,20^.

### Intermediate-risk patients

#### Primary endpoint

For early mortality, all eight studies except Gerfer et al. showed results favouring SU-AVR (OR: 0.425, CI (0.264–0.683)^21–28^. Figure 3). Most studies reported 1-year mortality (Sutureless/RDV: 1.7–8.4% vs. TAVR: 7-21.4%)^23–26^ and/or 2-year mortality (Sutureless/RDV: 4.9–8.4% vs. TAVR: 10.9%-33.8%)^22,24^. When all the intermediate risk studies were combined, 1-/2-year mortality was lower in the SU-AVR group (1-year mortality: SU-AVR: 7.39% vs. TAVR: 11.97%, 2-year mortality: SU-AVR: 9.68% vs. TAVR: 15.01%, HR: 0.579 (CI: 0.363–0.925), Log-rank *p* = .0014)(Figs. 5 and 6C).

#### Secondary endpoints

All studies reported PPI rates (OR: 0.906, CI (0.495–1.659), . 4C)^21–28^and meta-analysis confirms the wide spread of reported results, with an I² = 66.68 (*p* = .0038), showing substantial heterogeneity. Other reported postoperative complications included stroke (OR: 0.691 with CI (0.394–1.212)^21–28^ and AKI (OR: 0.824 with CI (0.313–2.170)^21–24,26,28^. Hospital length of stay (sutureless/RDV: 8.1–13 days vs. TAVR: 4.5–9.5 days) and ICU length of stay (sutureless/RDV: 1-5.9 days vs. TAVR 1-3.2 days) were longer in the SU-AVR group across all studies^22,24–28^.

#### Hemodynamic performance at discharge

Hemodynamic valve performance in terms of peak (DoM: 3.025 with 95% CI (-0.315–6.364) and mean TPG (DoM: 3.321 with 95% CI (-0.647–7.288) favored TAVR, although SU-AVR showed less PVL (OR: 0.125 with CI (0.064–0.247)^21–28^.

### High-risk patients

#### Primary endpoint

Of the three high surgical risk studies, early mortality (OR: 1.195 with CI (0.398–3.585) did not differ between the two procedures (. 3))^29,30^. SU-AVR group seems to be associated with a lower mortality (1-year mortality: SU-AVR: 4.2% vs. TAVR: 8.3%, 2-year mortality: SU-AVR: 8.4% vs. TAVR: 20.8%, HR: 0.400 (CI:0.130 − 0.1.233), Log-rank *p* = .0793)(Figs. 5 and 6D).

#### Secondary endpoints

Two out of three studies reported PPI rates (OR: 0.260, CI (0.051– 1.328), Fig. 4C)^29–31^. Other postoperative complications were only described by Bruno et al., including stroke (OR: 1.000, CI (0.060– 16.763), Fig. 4A), AKI (OR: 3.101, CI (0121– 79.205), Fig. 4B), ICU length of stay (sutureless/RDV: 3.1 days vs. TAVR: 2.1 days)^29^ and hospital length of stay (sutureless/RDV: 7.7–12 days vs. TAVR 8-9.4 days)^29,30^.

#### Hemodynamic performance at discharge

Postoperative hemodynamic performance was only reported by Bruno et al. with peak TPG (DoM: 3.6 with CI (0.184–7.016)) and mean TPG (DoM: 2.5 with CI (0.396–4.604)) and PVL (OR: 0.187 with CI (0.009–4.062), (Fig. 4D-F)^29^. No significant differences between PVL rates were observed, possibly due to a limited power in the high-risk group, while post-operative TPG were significantly lower for the patients that underwent TAVR.

## Discussion

Sutureless and rapid deployment valves have been compared to TAVR, though never in a RCT, as all RCTs involved mainly standard sutured valves in the surgical arm^32–37^. In absence of such RCTs, the information that can be extracted from propensity score matched comparative studies is still valuable, as they show results in a real world population.

The sutureless or rapid deployment valves used in the studies included in this analysis were the Perceval S valve and the Intuity valve. Although both valves often show very similar outcomes in terms of mortality and morbidity, the reduction of procedure times is larger for the Perceval valve whereas the Intuity shows better hemodynamic performance^38–41^. Likewise, there are differences in the valves that were used in the TAVR groups. The two main valve groups investigated were the balloon-expandable (BE) Sapien and self-expandable (SE) CoreValve/Evolut series. The Sapien series consist of the Sapien, Sapien XT and the Sapien 3 while Corevalve evolved to Evolut R and Evolut Pro, both exhibiting improved outcomes with every new generation^42^. Overall, the SE CoreValve series have shown higher rates of PPI which can be explained by the intrinsic radial forces required for self-expanding valve fixation^42–44^.

Most of the low-risk patients in our meta-analysis come from one registry: the GARY registry from Germany. Pooled analysis in this risk group confirms that TAVR outperforms SU-AVR in terms of early mortality, AKI, TPG, and shorter length of stay at the cost of more PPI and PVL. These favourable outcomes for TAVR can possibly be explained by the exclusively transfemoral approach in the GARY experience. Generally, the transfemoral approach has become the preferred access route as it has shown better immediate outcomes^45,46^. Of note, Vilalta et al. reported a surprisingly high early mortality (4.1%) in the sutureless group, which is higher than in most studies in the intermediate/high-risk groups. Long-term outcome data are scarce in this low-risk population. Vilalta et al., reported hazard ratio of 0.970 (CI 0.518–1.815), showing no difference between the two treatments.

These results are in line with the findings in the large RCTs comparing TAVR with SAVR in low-risk patients, in which TAVR was found to be non-inferior or even superior to SAVR in terms of the primary outcome. Despite the all-comer nature in the registries included in our meta-analysis, patient risk profile and outcomes were comparable to those in the RCTs, with STS scores around 2% and similar stroke and early mortality rates^34,37^. In this respect, the use of sutureless or RDV does not seem to present a particular advantage to conventional surgical valves.

In contrast with low-risk patients, intermediate risk patients seem to fare exceptionally well with SU-AVR, with a significantly lower early mortality rate as compared with TAVR in this meta-analysis. This benefit was confirmed in the studies reporting longer term follow-up. While the difference in hemodynamic performance of the implanted valves was similar to low-risk patients (higher TPG but less PVL for sutureless or RDV), there are conflicting results regarding stroke, AKI and PPI (some studies show less, equal or more stroke, AKI or PPI after TAVR). Chung et al. reported a remarkably high incidence of AKI (21%) after SU-AVR, however, this could potentially be due to the differences in definitions used by the different studies. Regarding the need for PPI, the higher incidence of PPI with TAVR was mainly driven by those studies using a larger proportion of SE valves^42^.

The lower early and medium-term mortality with SU-AVR in intermediate risk patients is in contrast with the findings in the large RCTs comparing TAVR with SAVR in this risk category. However, the weighted average STS risk score in our meta-analysis were at least as high (5.7%) as compared with the RCTs with BE (5.8%) and SE (4.4%) TAVR valves. Interestingly, early mortality rates were higher for TAVR in the meta-analysis as compared to the TAVR arms of the RCTs (weighted average: 4.9% vs. BE: 3% and SE: 0.5%, respectively), while early mortality was lower in the surgical arms of the SU-AVR meta-analysis as compared to the RCTs (weighted average: 2% vs. BE: 4.1% and SE: 1.3%, respectively)^33,36^. These differences suggest improved performance of SU-AVR of sutured valves in this patient population, as well as a poorer TAVR outcomes in daily practice as compared to the RCTs. Another contributing factor may be some degree of bias being introduced in the treatment selection in the registry studies, despite the propensity matching that has been applied. Indeed, it is conceivable that patient characteristics not included in the propensity matching may have driven the choice for SAVR rather than TAVR, or vice versa, in this patient population.

According to treatment guidelines, high-risk patients with severe AS should preferably be treated with transfemoral TAVR, when technically feasible. In our meta-analysis, only limited data is available regarding the comparison between SU-AVR and TAVR in this group. The studies from Bruno and Santarpino et al. are very similar, both using the SE Corevalve with 100% transfemoral approach in TAVR patients. Within the few outcomes reported by both studies, there are noticeable differences in the in-hospital mortality and hospital LOS. Another important difference is in the need for PPI in the TAVR groups, which is almost three times as high for Bruno et al. compared to Santarpino et al. A possible explanation might be that the latter has a larger patient population, with a longer inclusion time, resulting in a better learning curve to optimize outcome^47^. While the presented data hardly permit one to make solid conclusions regarding the role of SU-AVR versus TAVR in this high-risk population, these studies confirm that SU-AVR may be a good alternative to conventional SAVR for these patients, especially when TAVR is contraindicated. Shorter XCT and procedure times are of particular benefit in an often frail and aged population with multiple comorbidities.

### Limitations

The present systematic review and meta-analysis has several limitations that are inherent to the nature of the studies included. Reconstructed IPD rely on the level of information given, e.g., number at risk and the quality of the graphics. When lines are tangled, it can be difficult to separate out the KM-curves for digitizing. Moreover, the underlying algorithm presumes uniform censoring, an assumption that may not hold in all studies. The original authors have also indicated the need for subsequent research to investigate the variability of test statistics (even though their limited investigations showed promising results). In general, propensity score matching across studies tries to match patient characteristics. However, even when applying a correct statistical methodology, propensity matching cannot fully correct for the heart team decision. Not all discussed variables, such as patient frailty, mobility, mental health etc., are systematically reported and hence not included in the model. Overall, even after matching, the TAVR population was a slightly older and of higher risk than the SU-AVR group. This difference is not negligeable and could have an influence on the outcomes presented in this meta-analysis. Truly balanced (randomized) analyses should include patients who fully qualify for both treatment options before treatment selection. Moreover, older studies included differences in learning curve, mainly for TAVR, but also for SU-AVR. Another aspect that increases the difficulty to interpret the results is the many different valve types used in the included studies. Furthermore, valve designs are continuously changing (mainly of the TAVR valves) and sometimes implantation techniques were adapted (location of suture of the Perceval valve to avoid PPI and a change in sizing strategy to prevent oversizing). Moreover, the included studies contain many rather small sample sizes. Specifically in the low- and high-risk groups, the number of included studies is limited and have a noticeable disbalance in sample size within each group. Additionally, for the low- and high-risk groups, the length of follow-up is limited compared to the intermediate risk group. All are factors that warrant attention to the interpretation of results and comparison with the intermediate risk group. Ideally a large, randomized trial comparing SU-AVR with TAVR (ideally in low to intermediate surgical risk patients) should be performed in the future.

When combining all risk groups, our meta-analysis reveals no difference in early mortality between SU-AVR and TAVR, however SU-AVR was associated with an increased survival at 1- and 2-years. Stroke and AKI rates were not different between the two procedures. SU-AVR resulted in lower rates of PPI and significant paravalvular leakage, while TAVR patients had significantly better peak and mean post-operative TPG and a shorter hospital stay. On the basis of this analysis, we can also conclude that both approaches are associated with a different pattern of complications, something that may guide the choice for the individual patient, confirming again the importance of the Heart Team and individual tailored treatment strategies.

## Conclusion

Based on our meta-analysis, which is based on a limited number of non-randomized studies, SU-AVR is not inferior to and possible superior to TAVR for certain outcomes, especially in the intermediate risk patients. Until stronger evidence is available SU-AVR should be discussed as a potential valid treatment in patients with severe AS that are discussed for TAVR. Randomized clinical trials, ideally with long-term follow-up are essential to exactly determine the ideal patients for both procedures.

## Supplementary Information

Below is the link to the electronic supplementary material.

Supplementary Material 1

## Data Availability

All data generated or analyzed during this study are available from the corresponding authors on reasonable request.

## Abbreviations

AKI: Acute kidney injury
AS: Aortic valve stenosis
AXC: Aortic cross clamp
BE: Balloon-expandable
CBP: Cardiopulmonary bypass
CVVH: Continuous venovenous hemofiltration
DoM: Difference of the means
ECC: Extra corporeal circulation
HR: Hazard ratio
ICU: Intensive care unit
IPD: Individual person data
KM: Kaplan-Meier
Mi-AVR: Minimally invasive aortic valve replacement
OR: Odds ratio
PARTNER trial: Placement of aortic transcatheter valves trial
PCI: Percutaneous coronary intervention
PPI: Permanent pacemaker implantation
PVL: Paravalvular leakage
RCT: Randomized controlled trial
RDV: Rapid deployment valve
ROBINS-I: Risk of bias in non-randomized studies of interventions
SAVR: Surgical aortic valve replacement
SE: Self-expandable
STS: Society of thoracic surgeons
STS PROM: Society of thoracic surgeons predicted risk of mortality
SU-AVR: Sutureless aortic valve replacement
TAVR: Transcatheter aortic valve replacement
TPG: Transprosthetic gradient
