# Supplementary material for: Sutureless versus transcatheter valves for the treatment of aortic valve stenosis: a systematic review and meta-analysis
Source: Sci Rep. 2025 Oct 3;15:34540. doi: 10.1038/s41598-025-17857-7 (PMC12494754; doi:10.1038/s41598-025-17857-7)
Supplement: Supplementary file 1 — Supplementary Material 1 [file 41598_2025_17857_MOESM1_ESM.docx]

**Supplemental figures:**

Supplemental figure 1: Funnel plots visualizing the publication bias.
AKI: acute kidney injury, PVL: paravalvular leakage.

**
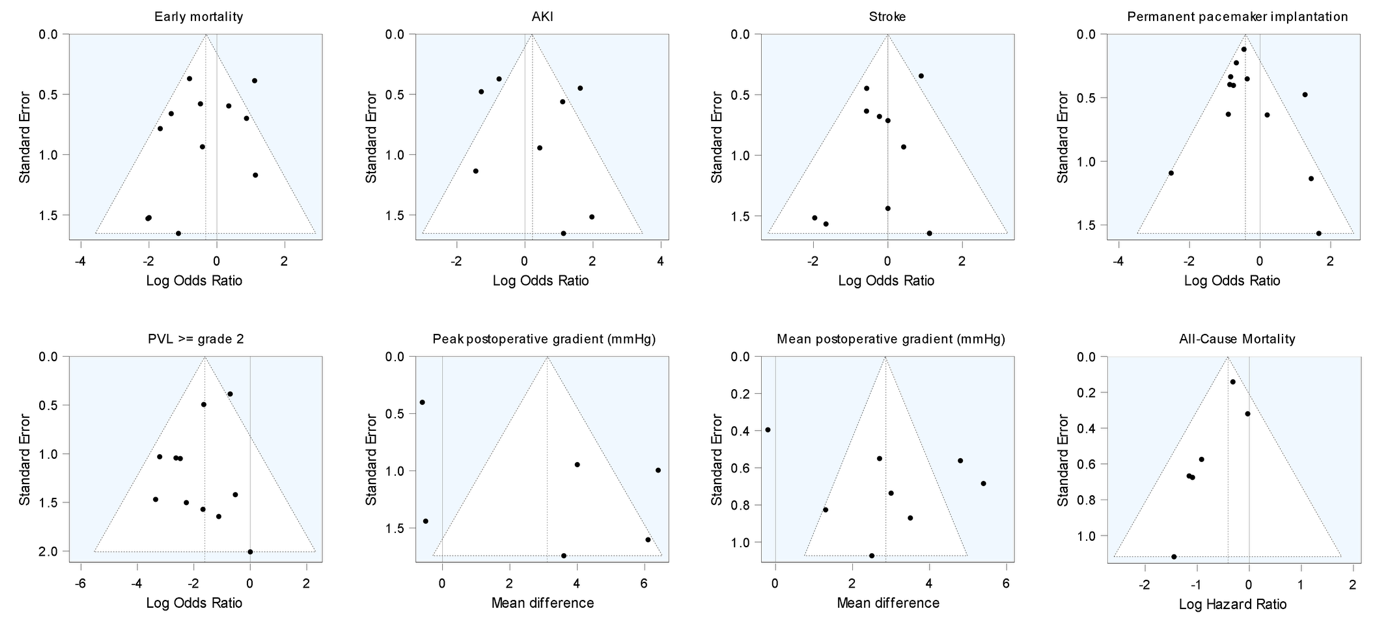
**
